# Real-Life Testing of the Prescription Opioid Misuse Index in French Primary Care

**DOI:** 10.3390/ijerph192214845

**Published:** 2022-11-11

**Authors:** Catherine Laporte, Frédéric Fortin, Julie Dupouy, Aurélie Quirin, Bruno Pereira, Chouki Chenaf, Jessica Delorme, Christine Maynié-François, Cédric Rat, Jordan Birebent, Jacques Rambaud, Christian Duale, Nicolas Kerckhove, Noémie Delage, Nicolas Authier

**Affiliations:** 1 Clermont Auvergne Institut Pascal (INP), CNRS, Centre Hospitalo-Universitaire (CHU) Clermont-Ferrand, Université Clermont Auvergne, F-63000 Clermont-Ferrand, France; 2 Unite Mixte de Recherche (UMR) 1295 Inserm, Université Toulouse III, F-31000 Toulouse, France; 3 Maison de Santé Pluriprofessionelle Universitaire de Pins Justaret, F-31860 Pins Justaret, France; 4 Unité de Biostatistiques, Direction de la Recherche Clinique et de l’Innovation, CHU Clermont-Ferrand, F-63000 Clermont-Ferrand, France; 5 CHU Clermont-Ferrand, Service de Pharmacologie Médicale, Centre d’Addictovigilance et Pharmacovigilance, Centre Evaluation et Traitement de la Douleur, Inserm, Neuro-Dol, Université Clermont Auvergne, F-63000 Clermont-Ferrand, France; 6 UMR CNRS 5558, Laboratoire de Biométrie et Biologie Évolutive (LBBE), Équipe Evaluation et Modélisation des Effets Thérapeutiques (EMET), Université Claude Bernard Lyon 1, F-69100 Lyon, France; 7 Département de Médecine Générale, Université de Nantes-INSERM U1302/CNRS EMR6001-Équipe 2, F-44035 Nantes, France; 8 Département de Médecine Générale, Université Montpellier, F-34090 Montpellier, France

**Keywords:** primary care, general medicine, pain, analgesics, misuse, screening

## Abstract

Analgesic opioid (AO) misuse by patients ranges from 0% to 50%. General practitioners are the first prescribers of AO. Our objective was to validate the Prescription Opioid Misuse Index (POMI) in primary care. We conducted a psychometric study in patients with chronic pain who had been taking AOs for at least 3 months and were followed in general practice. Patients responded to the POMI at inclusion and after 2 weeks. The reference used was the DSM-V. Sixty-nine GPs included 160 patients (87 women, 54.4%), mean age 56.4 ± 15.2 years. The total POMI score was 1.50 ± 1.27, and 73/160 (45.6.0%) had a score ≥ 2 (misuse threshold). Internal validity was measured with the Kuder–Richardson coefficient, which was 0.44. Correlations between each item and the total score ranged from 0.06 to 0.35. Test–retest reliability was determined from 145 patients: Lin’s concordance coefficient was 0.57 [0.46, 0.68]. Correlation with the DSM-V (Spearman’s coefficient) was 0.52. The POMI does not have sufficient psychometric properties to be recommended as a tool to identify the misuse of AOs in primary care. This study clearly showed that there is a need to create a monitoring tool specific to primary care.

## 1. Introduction

Chronic pain is a major issue in terms of the impact on the individual’s quality of life [1] and on society. High costs are generated by delays in treatment and care [2]. The prevalence of chronic pain in the general population varies from 10.1 to 55.2% [3]. General practitioners (GP) are on the frontline for the treatment of pain: pain represents 43% of the reasons for consultation, 24% of which are for chronic pain [4]. More than half of patients are exclusively cared for by their GP [5]. The others are followed up in pain assessment and treatment centers [6].

In 2015, nearly one in five French people (17.1%) underwent opioid treatment [7]. The risk of opioid use disorder secondary to opioid analgesics in patients with chronic pain varies from 0% to 50% [8]. American recommendations advocate the periodic surveillance of opioid use disorder when chronic opioid analgesics are prescribed, depending on the patient’s risk factors [9]. The French “Limoges” recommendations also mention performing a systematic search for signs of psychological dependence during treatment and state that treatment with strong opioids should be stopped in the event of misuse, abuse, or addiction [10]. They recommend that signs of misuse or psychological dependence (characterized by craving) should be sought at each examination to verify that strong opioids are being correctly used in chronic osteo-articular pain. Identifying misuse is a way of optimizing the benefit/risk ratio [11].

The difficulty establishing the prevalence of misuse results from the lack of standardization of studies and the lack of consensus in the use of assessment tools. Several tools are available internationally [9,11]. The only diagnostic criteria available are those of the DSM-V (Fifth Edition of the Diagnostic and Statistical Manual of Mental Disorders) [12] and the ICD-10 (International Classification of Diseases) [13], which notably overestimate the prevalence because of the frequent presence of tolerance and withdrawal signs without misuse or addiction [11]. Today, no screening tool has been validated in France for primary care, but the authors recently validated the POMI scale in French to screen patients specifically followed in pain clinics and presenting misuse behavior during their opioid analgesic treatment (POMI5F) [14].

In 2015, treatment was initiated by a GP in 59.1% of cases for weak opioids and 62.9% of cases for strong opioids and by a hospital doctor for 20.1% and 21% respectively [7]. Currently, we do not have such a tool in primary care. A tool validated in French would make it possible to standardize screening practices and ensure safe prescription both from the point of view of the doctor and of the patient. Furthermore, the lack of a validated tool in French is an obstacle to the development of true pharmaco-epidemiological studies on the prevalence of opioid misuse. The originality of this study is the assessment of the clinical relevance of the French transcultural validation of the POMI scale in primary care to ensure appropriate and relevant use by all health professionals and to allow the large-scale screening of misuse behavior of analgesic opioids.

## 2. Objectives

### 2.1. Main Objective

The aim of the study was to validate the French version of the Prescription Opioid Misuse Index (POMI) in patients with chronic pain (neuropathic, dysfunctional, excess of nociception) in a general practice setting.

### 2.2. Secondary Objective

- To study the profile of patients who misuse opioid analgesics
- To compare the results of this study with those of a previous study of patients followed in a pain clinic [14]

## 3. Method

We conducted a prospective, observational and multicenter psychometric study to cross-culturally validate an opioid analgesic misuse screening scale (POMI) in patients with chronic pain in primary care in France. The study was registered on Clinicaltrial.gov: NCT05431985.

### 3.1. Recruitment

All GPs working in general practices in four areas of France (Auvergne, Rhône-Alpes, Occitanie, and Pays de Loire) were personally invited by email to take part in the trial. GPs who had undergone specialized training in addiction treatment (e.g., university degree, qualification, university course) were not included. GPs included patients regardless of the motive for consultation.

### 3.2. Inclusion

#### 3.2.1. Inclusion Criteria

- Patients aged 18 years and over
- Patients with chronic pain (neuropathic, dysfunctional, excess nociception) for at least 6 months
- Patients with a GP prescription for at least one opioid analgesic drug taken daily for at least the previous 3 months
- Patients registered with the French insurance system

#### 3.2.2. Exclusion Criteria

- Discontinuation of opioid prescriptions on the test step day (no retest possible)
- Patients in the process of withdrawal (risk of cessation during the retest step)
- Patients unable to complete the questionnaire alone
- Patients monitored by a pain clinic or addiction center
- Patients with ongoing cancer
- Patients who refused to participate

### 3.3. Measured Variables

#### 3.3.1. Outcomes: Prescription Opioid Misuse Index (POMI) [15]

The Prescription Opioid Misuse Index (POMI) was developed in the United States to assess oxycodone misuse. This scale was validated in 137 subjects recruited from pain clinics, addiction treatment programs, jails, or private medical practice [15]. The POMI is an 8-point self-assessment scale. Each point is rated as 0 (absence) or 1 (presence), and the sum of the points is used to calculate a total score (between 0 and 8): a score of 2 or above is considered a positive and indicates misuse. The sensitivity and specificity are 82% and 92% respectively. Internal consistency, measured with the Kuder–Richardson coefficient, is 0.848.

Although this scale was validated for oxycodone, the evaluation also applies to other opioids (morphine, tramadol, codeine, opium powder). Misuse does not differ between categories of opioids, and no differentiation between categories is proposed by other validated scales. We also chose this scale because of its reliable scoring and performance, which are two important criteria for the broad use of such a tool in primary care.

Knisely et al. [15] found that correlations were lowest for Items 4 and 5 and that the Chronbach alpha was highest with Items 4 and 5 removed; therefore, we dropped these two items.

#### 3.3.2. Assessment

GPs completed the DSM-V questionnaire [12]. As other screening tools have not been validated in French, the DSM-V diagnostic criteria were used as the gold standard, although they are less suited to addiction to medications, as they overestimate the notions of tolerance and withdrawal.

#### 3.3.3. Measurements

GPs asked patients about their medical history; type of pain (neuropathic, dysfunctional, excess of nociception); duration of pain (6–12 months, 1–5 years, >5 years); pain severity at its “worst” and “average”, measured with a numeric rating scale (NRS) (no pain = 0 to unbearable pain = 10); treatment (analgesia and any other). They collected the type of analgesia used: analgesic opioids, non-steroidal anti-inflammatory drugs (NSAIDs), paracetamol, nefopam, and antimigraine drugs. The average daily dosage and duration of treatment (3–6 months, 6–12 months, 1–5 years, >5 years) were collected.

GPs asked patients about sociodemographic data (age, sex, family status, professional status); medical and family medical history; history of psychiatric disorders; and substance use and abuse.

### 3.4. Study schedule

#### 3.4.1. Translation of the POMI scale in French

**Figure 1:**
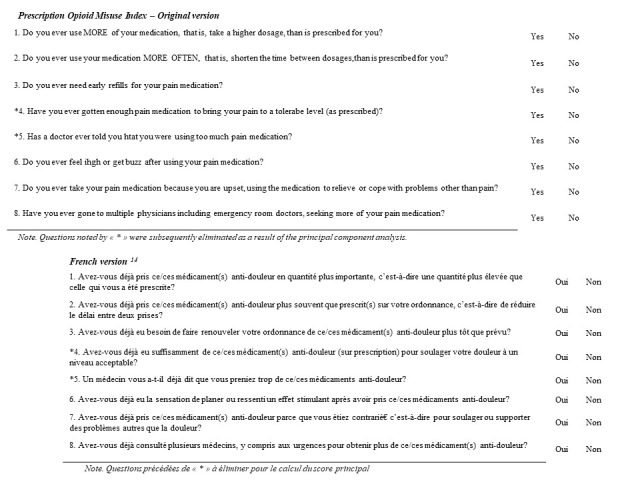
Prescription opioid misuse index, English and French version.

The translation of the POMI scale to French was conducted according to the recommended cross-cultural adaptation process [16]: (translation (English–French), adaptation of the different translations, back-translation (French–English); comparison of the back-translation and original POMI, and the acceptability of the final version. This was described in a prior article about patients in a pain clinic [14] (Figure 1).

#### 3.4.2. Recruitment

GPs were recruited by general medicine academic departments or research networks and patients were recruited from 16 January 2017 to 3 March 2019. GPs then received a brief e-learning training course (8 min) on the problem of investigating and setting up the study.

#### 3.4.3. Test

##### Inclusion Test

Each GP was asked to include three consecutive patients regardless of the reason for consultation if they fulfilled the inclusion criteria, without anticipating possible misuse, over a period of 24 months. Patients were given an information letter summarizing the goals of the study. GPs informed patients that the data would be anonymized and strictly confidential and that that their decision to take part in the study or not would not impact their treatment in any way.

At inclusion (test phase), GPs performed a clinical examination and assessed items of the DSM-V diagnostic criteria. The patient replied to the POMI questionnaire without the help of the GP. Completion time was around 15 min.

At the end of the consultation, the GP gave the patient the retest questionnaire, containing only the POMI scale and a pre-stamped envelope.

##### Retest Step

The retest step was conducted within 2 to 4 weeks after the test step. Patients sent the POMI scale completed it at home, then took it back to the coordination center. If necessary, a reminder was sent 10 days after the theoretical return date.

### 3.5. Statistics

Sample size estimation was fixed according to the COSMIN recommendations [17]. Accordingly, it was decided to include a minimum of 150 patients to analyze the consistency and internal validity, reproducibility, accuracy, and external validity with satisfactory statistical power. More precisely, rules of thumb [18] for the number of subjects needed to determine internal consistency vary from 4 to 10 subjects per variable, with a minimum number of 100 subjects to ensure the stability of the variance–covariance matrix. For reproducibility, at least 50 patients were needed to highlight a positive rating for reliability of at least 0.70. To recruit 150 patients for a total duration of inclusion of 12 months, four academic departments of general practice were asked to participate, and each GP was asked to include three patients. Each academic department therefore had to include 13 GPs.

The statistical analyses performed in this study were those usually performed in studies to validate scales [18]. In addition to descriptive statistics, the following psychometric properties of the POMI scale were explored using Stata Software (version 15, StataCorp, College Station, TX, USA): (i) acceptability and content validity: data quality was considered satisfactory if more than 95% of the scale data were fully computable. Floor and ceiling effects were analyzed. (ii) Internal consistency was determined with the Kuder–Richardson coefficient (minimum accepted value: 0.70), item–rest correlation (i.e., the correlation between the reported item and the total score excluding the reported item), and the item–total correlation corrected for overlap (criterion value: ≥0.30). (iii) Reproducibility: Lin’s concordance coefficient was used to determine the test–retest reliability for continuous outcomes, and Kappa’s concordance coefficient was estimated for categorical data. Values ≥ 0.70 were deemed satisfactory. (iv) Hypothesis testing: For convergent validity, relationships between DSM-V and POMI scale scores were evaluated with correlation coefficients (Pearson or Spearman, according to the statistical distribution) and ROC analysis followed by the estimation of the Youden and Liu indices to determine the best threshold of POMI to discriminate regarding those the DSM-V categorized as >3.

Continuous variables are presented as means and standard deviations or medians and inter-quartiles. To compare patient characteristics according to DMS-V results (<4/≥4) and to compare patient characteristics from this study (primary care) and the pain-clinic study, Chi-squared and Fisher’s exact tests were used for categorical data and Student t-tests or Mann–Whitney tests were applied for continuous variables. Homoscedasticity was evaluated with the Fisher–Snedecor test. All statistical tests were performed for a two-tailed type I error at 5%.

Then, analyses were completed by factorial analysis to compare the characteristics of the pain-clinic-study patients and the primary-care patients. More precisely, mixed data factorial analysis, combining categorical and continuous data, was conducted with the following variables: age, sex, employment, pain type, and treatment. These variables were chosen according to the univariate results, their clinical relevance, and their statistical distribution (variables always present or always absent were not considered). Group (participants in the pain clinic study and participants in the primary-care study) was treated as an illustrative variable. Only individuals without missing data were included in the factorial analysis. This exploratory method was used to summarize the relationships between variables and to detect the underlying structure of the data, i.e., patterns of patients. A sensitivity analysis was conducted to study the impact of missing data on results comparing the samples with and without missing data for the main patient characteristics.

## 4. Results

We recruited 69 GPs, 68 of whom included at least one patient. They were 42.8 years old [min 29–max 65], lived mostly in urban areas (78.0%), and 34/68 (50.0%) were female.

From January 2017 to March 2019, the GPs included 160 patients, 87/160 (54.4%) were female, and the mean age was 56.4 ± 15.2 years. Descriptive statistics are shown in Table 1.

Type of pain was mainly nociplastic (58.8%, *n* = 98), and 55.6% (*n* = 89) of patients had had pain for at least 5 years. The main types of opioid analgesia used were tramadol (40.1%, *n* = 65), followed by codeine (21.9%, *n* = 35), oxycodone (15.0%, *n* = 24), and morphine (14.7%, *n* = 23). The frequently used non-opioid analgesia was acetaminophen (53.1%, *n* = 85) and NSAID (11.3%, *n* = 18).

### 4.1. Acceptability and Content Validity

**Figure 2:**
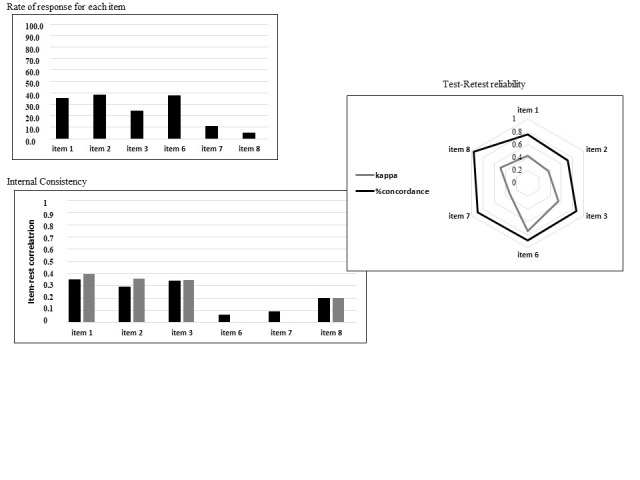
Psychometric properties.

The results for the data quality and acceptability of the POMI scale are shown in Figure 2. Fully computable data were obtained for the entire sample (*n* = 160). The rate of patients who responded positively to individual items was lowest for Items 7 and 8 (10.5% and 5%, respectively) and highest for Items 2 and 6 (38.1% and 37.5%, respectively).

### 4.2. Internal Consistency

Figure 2 displays data on the internal consistency of the POMI scale. The Kuder–Richardson coefficient of reliability for the POMI, calculated as reported by Knisely et al., was 0.44, and 73/160 (45.6%) patients had a score ≥ 2. The item–rest correlation ranged from 0.058 (Item 6) to 0.348 (Item 1). When Items 6 and 7 were removed, the Kuder–Richardson coefficient increased to 0.54, with item–rest correlation coefficients ranging from 0.20 (item 8) to 0.40 (item 1).

### 4.3. Test–Retest

Test–retest reliability was determined in 140 patients. For the POMI total score, Lin’s concordance coefficient was 0.57 [0.46, 0.68], with 1.50 ± 1.27 at the test step and 1.01 ± 1.16 at retest. When the POMI score was dichotomized by a cut-off of 2, Kappa’s Cohen concordance coefficient was 0.42, with 72.9% agreement.

For the POMI score excluding Items 6 and 7, Lin’s concordance coefficient was 0.55 [0.44, 0.66], with 1.03 ± 1.08 for the test step and 0.65 ± 0.96 for retest. When the POMI score was dichotomized by a cut-off of 2, Kappa’s Cohen concordance coefficient was 0.38, with 78.6% agreement.

### 4.4. Construct Validity

The correlation between the POMI score and DSM-V was r = 0.52 (*p* < 0.001). When POMI and DSM-V were respectively categorized as <2, >2 and <4, 4, or 5, ≥6, 82/160 patients had a score of <2 and <4, and 28/160 had ≥2 and ≥4 as expected, whereas 50/160 patients had ≥2 and <4 or <2 and ≥4.

The item-by-item analysis showed that items 1, 2, 3, 6, and 8 were correlated with the DSM-V, whereas item 7 was not.

### 4.5. Comparison between Participants According to DSM-Vscore

As the psychometric properties of thesholds had not been assessed, we compared DSM-V scores between patients with a low addiction score (>4) 127/160 (79.4%) and a moderate to severe addiction (≥4) 33/160 (20.6%), Table 2.

Some sociodemographic differences were found between these groups: patients with moderate or severe addiction were younger (46.9 ± 11.9 vs. 58.9 ± 15.1, *p* = 0.001), more often single (*p* = 0.03), more often inactive, or in a situation of disability (*p* = 0.01). There was no difference in type of pain and treatment according to the addiction score.

### 4.6. Comparison between Patients of Study in the Pain Clinic and Primary-Care Studies

Patients recruited in primary-care centers were older (56.4 ± 15.2 vs. 50.2 ± 11.8, *p* < 0.001), less often in a relationship (92 (57.5%) vs. 103 (73.1%), *p* < 0.001). They more often had nociceptive pain (83 (51.9%) vs. 33 (21.4%), *p* < 0.001), higher pain intensity in the last 24 h (7.4 ± 1.9 vs. 6.8 ± 1.9, *p* < 0.01), and they have been in pain for less time. Many of them took codeine (35 (21.91%) vs. 17 (11.0%), *p* < 0.01) and paracetamol (85 (53.1) vs. 59 (38.3), *p* < 0.001) (Table 1).

**Figure 3:**
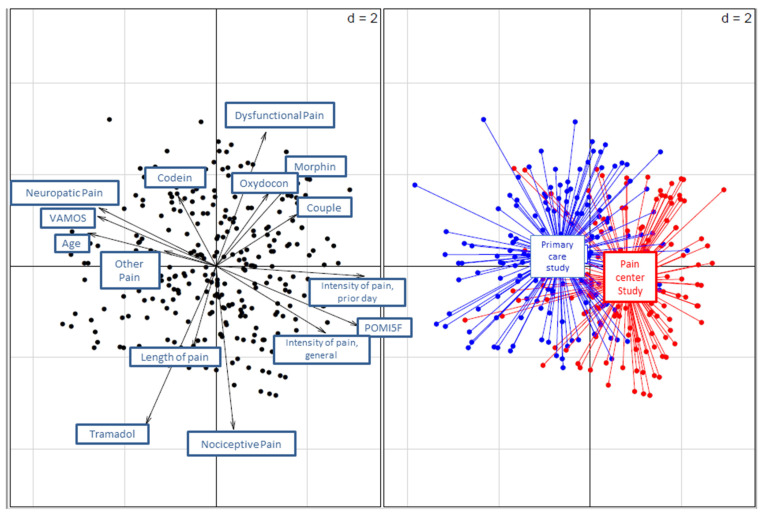
Distribution of the variables in the samples of the two studies: pain center and primary care.

For the factorial analysis, 21 out of 314 (6.7%) patients were removed because of missing data, and 293 were retained. Vector analysis identified that the variables were distributed differently in the samples of the two studies (Figure 3). The two samples did not differ significantly in terms of any variables selected for analysis, except for previous-day pain intensity.

## 5. Discussion

### 5.1. Main Results

This is the first validity study of a European-French version of the Prescription Opioid Misuse Index (POMI) in patients with chronic pain (neuropathic, dysfunctional, excess of nociception) followed in a primary-care setting. We found that the psychometric properties of the POMI were insufficient to be used in primary care. Internal consistency measured with the Kuder–Richardson coefficient in 160 patients was moderate (0.44); almost half of the sample (45.6%) showed misuse (score ≥ 2). The item–rest correlation for the total score ranged from 0.06 to 0.35. Test–retest reliability in 140 patients was moderate (0.57 [0.46, 0.68]). The POMI score was moderately correlated (r = 0.52) with the DSM-V score, which was the reference.

### 5.2. Comparison with the Literature

Our study sample was similar to the French population of opioid users described in 2019 by ANSM [7], although our sample included more women (54.4%), who were slightly older (56.4 ± 15.2 years vs. 50.0 [7]), and were mostly weak opioid users (73.9%) vs. strong opioid users (32.8%) (with 40.1% for tramadol).

To validate the psychometric quality of a test, a Cronbach alpha of 0.7 is expected [18,19]. The Cronbach alpha of the initial study was 0.84 [15] and that of the pain clinic study was 0.71 [14]. In this primary care study, the Cronbach alpha was only 0.44. Reproducibility was also lower than for the pain clinic study (Lin’s concordance coefficient 0.57 [0.46, 0.68] vs. 0.65 [0.55, 0.67]. The correlation between the pain-clinic-study score and DSM-V was slightly higher (r = 0.52 (*p* < 0.001) vs. r = 0.45, *p* < 0.001).

Classified according to DSM-V score, non-misusers were older and more often in a relationship, but there were no major differences in terms of pain (type or duration) or treatment (opioids or non-opioids). On the other hand, the profiles of the patients differed between the samples of the two studies. Patients recruited in primary-care centers were older, more often had nociceptive pain, had higher pain intensities in the last 24 h, and have been in pain for less time. They more often took codeine and paracetamol but less often gabapentin. These results suggest that it is the difference between samples that led to the weaker psychometric properties of the tool when used in primary care. A tool specific to this population should therefore be developed.

Over the past 10 years, GP consultations have been enriched by the availability of a multitude of tools [20]. Around 13,500 [21] medical decision support tools have been developed, including screening tests. These tests are little-known and little-used in practice. A recent study asked French GPs about the ten tests that correspond to the most frequent reasons for consultation: they knew only six of them and only used four [22]. The GPs who knew the tests but did not use them reported doubting their usefulness for patient management [22]. Indeed, these tests are not always validated by ad hoc studies, and when they are, the methodology is often imperfect [23,24]. Regardless of the methodology, they are mostly validated in hospitals and in English, which poses the problem of generalization to all patients.

Lack of time and training were also cited as barriers to using a screening tool [22]. The POMI scale is short and concise, which facilitates its administration by physicians in daily use in clinical practice.

### 5.3. Strengths and Weaknesses

The recruitment of patients in a GP practice is sometimes complicated. Not all GPs included patients, and those who did were able to choose the patients they included. During the test phase, the questionnaires were completed by patients in front of a clinician, and during the retest step, the same version was completed at home by patients. The test–retest reliability may have been affected by social desirability bias [25]. We used the DSM-V as a comparator for the POMI, although it is not the gold-standard tool for the identification of opioid misuse.

## 6. Conclusions

GPs are the first prescribers of opioids. Misuse, which is a broader concept than addiction, can be assessed at two stages: before the first prescription to identify the risk of developing misuse and during treatment to identify misuse. A misuse identification tool adapted to primary care would facilitate GP awareness of the two stages, as well as the correct use of opioid analgesics. This study clearly showed that there is a need to create a monitoring tool specific to primary care, that ensures the safe prescription of opioid analgesics by standardized and quick, regular monitoring in agreement with national and international recommendations.

## Figures and Tables

**Table 1:** Description of the populations of the studies.

	Study Sample	Sample from the Pain Clincic Study	
	Total (*n* = 160)	Total (*n* = 154)	*p*
Female sex *n* (%)	87 (54.4)	98 (63.6)	0.1
Age, mean ± SD	56.4± 15.2	50.2 ± 11.8	<0.001
In relationship, *n* (%)	92 (57.5)	103 (73.1)	0.005
Socioprofessional situation			
Active	52 (34.0)	na	
Inactive	9 (5.9)	na	
Disability	46 (30.1)	na	
Retirement	46 (30.1)	na	
Socioprofessional Category			
Senior	12 (8.6)	na	
Employee	89 (64.0)	na	
Independent	38 (27.3)	na	
Type of pain, *n* (%)			
Nociplastic (dysfunctional)	94 (58.8)	94 (61.0)	0.68
Nociceptive	83 (51.9)	33 (21.4)	<0.001
Neuropathic	74 (46.3)	66 (42.9)	0.55
Pain duration, *n* (%)			
6–12 months	11 (6.9)	1 (0.7)	0.02
1–5 years	60 (37.5)	58 (38.1)	
≥5 years	89 (55.6)	93 (61.2)	
Pain intensity (24 h)			
Total score (/10), mean ± SD	6.8 ± 1.9	7.4 ± 1.9	<0.01
Intensity < 3/10, *n* (%)	4 (2.5)	5(3.3)	<0.01
Intensity from 3/10 to 6/10, *n* (%)	56 (35)	27(17.5)	
Intensity ≥ 7/10, *n* (%)	100 (62.5)	122(79.2)	
Pain intensity (general)			
Total score (/10), mean ± SD	6.1 ± 1.8	6.3 ± 1.9	0.17
Intensity < 3/10, *n* (%)	2 (1.3)	6 (3.9)	0.02
Intensity from 3/10 to 6/10, *n* (%)	97 (60.6)	71 (46.1)	
Intensity ≥ 7/10, *n* (%)	61 (38.1)	77 (50.0)	
% relief with treatment, mean ± SD	57.2 ±22.1	50.5 ± 24.6	0.01
DSM-V score			
Total score, mean ± SD	1.9 ± 2.4	1.7 ± 2.0	0.27
≤ 3: mild, *n* (%)	127(79.3)	132 (86.3)	0.23
4–5: moderate, *n* (%)	23(14.4)	13 (8.5)	
≥ 6: severe, *n* (%)	10(6.3)	8 (5.3)	
Opioid treatment used, *n* (%)			
Morphine	24 (15.0)	29 (18.8)	0,37
Fentanyl	4 (2.5)	9 (5.8)	0.14
Oxycodone	24 (15)	34 (22.1)	0.11
Hydromorphone	1 (0.63)	0 (0.0)	1,00
Tramadol	65 (40.1)	59 (38.3)	0.68
Codeine	35 (21.9)	17 (11.0)	0.01
Dihydrocodeine	0 (0.0)	0 (0.0)	
Opium	19 (11.9)	14 (9.1)	0.42
Concomitant analgesic treatments, *n* (%)			
Acetaminophen	85 (53.1)	59 (38.3)	<0.001
NSAIDs	18 (11.3)	10 (6.5)	0.14
Corticoids	3 (1.9)	3 (2.0)	0.96
Gabapentin	6 (3.8)	15 (9.7)	0.03
Pregabalin	14 (8.8)	23 (14.9)	0.09
Nefopam	3 (1.9)	3 (2.0)	1.0
Triptan	4 (2.5)	0 (0.0)	0.12
Amitriptyline	16 (10.0)	25 (16.2)	0.1

DSM-V: Fifth Edition of the Diagnostic and Statistical Manual of Mental Disorders; NRS: numeric rating scale; NSAIDs: non-steroidal anti-inflammatory drugs; SD: standard deviation, na: not available.

**Table 2:** Comparison of misusing and non-misusing patients according to their dependence score measured with the DSM-V.

	Dependance Score DSM-V		
	<4 (*n* = 127)	>=4 (*n* = 33)	
	*n* (%)	*n* (%)	*p*
Female, *n* (%)	66 (52.0)	21 (63.6)	0.2
Age, mean ± SD	58.9 ± 15.1	46.9 ± 11.9	0.001
Personal situation			
In relationship, *n* (%)	76 (59.8)	16 (48.5)	0.03
Single, *n* (%)	12 (9.5)	9 (27.3)	
Widowed, *n* (%)	15 (11.8)	1 (3.0)	
Divorced, *n* (%)	24 (18.9)	7 (21.2)	
Socioprofessional situation			
Active	38 (31.2)	14 (45.2)	0.01
Inactive	6 (4.9)	3 (9.7)	
Disability	34 (27.9)	12 (38.7)	
Retirement	44 (36.1)	2 (6.5)	
Socioprofessional Category			
Senior	11 (10.1)	1 (3.3)	0.11
Employee	65 (59.6)	24 (80.0)	
Independent	33 (30.3)	5 (16.7)	
Type of pain, *n* (%)			
Nociplastic (dysfunctional) *	70 (55.1)	27 (72.7)	0.06
Nociceptive **	68 (53.54)	15 (45.5)	0.4
Neuropathic ***	60 (47.2)	14 (42.2)	0.62
Pain duration, *n* (%)			
6–12 months	9 (7.1)	2 (6.1)	0.96
1–5 years	48 (37.8)	12 (36.6)	
≥5 years	70 (55.1)	19 (57.6)	
Pain intensity (24 h)			
Total score (/10), mean ± SD	6.8 ± 1.8	6.9 ± 2.1	0.24
Intensity < 3/10, *n* (%)	2 (1.6)	2 (6.1)	0.3
Intensity from 3/10 to 6/10, *n* (%)	46 (36.2)	10 (30.3)	
Intensity ≥ 7/10, *n* (%)	79 (62.2)	21 (63.6)	
Pain intensity (general)			
Total score (/10), mean ± SD	6.0 ± 1.8	6.2 ± 1.7	0.23
Intensity < 3/10, *n* (%)	2 (1.6)	0 (0.0)	0.76
Intensity from 3/10 to 6/10, *n* (%)	77 (60.6)	20 (60.6)	
Intensity ≥ 7/10, *n* (%)	48 (37.8)	13 (39.4)	
% relief of treatment, means ± SD	56.1 ± 22.5	61.4 ± 20.0	0.25
Opioid treatment used, *n* (%)			
Morphine	21 (16.5)	3 (9.1)	0.29
Fentanyl	2 (1.57)	2 (6.1)	0.14
Oxycodone	22 (17.3)	2 (6.1)	0.11
Hydromorphone	1 (0.79)	0 (0.0)	1,00
Tramadol	49 (38.6)	19 (57.6)	0.05
Codeine	31 (24.4)	6 (18.1)	0.45
Dihydrocodeine	0 (0.0)	0 (0.0)	
Opium	16 (12.6)	6 (18.2)	0.41
Concomitant analgesic treatments, *n* (%)			
Acetaminophen	69 (54.3)	17 (51.5)	0.55
NSAIDs	14 (11.0)	4 (12.1)	0.86
Corticoids	3 (2.4)	0 (0.0)	0.37
Nefopam	2 (1.6)	1 (3.0)	0.58
Triptan	2 (1.6)	2 (6.1)	0.14
Gabapentin	6 (4.7)	0 (0.0)	0.20
Pregabalin	12 (9.5)	2 (6.1)	0.54
Amitriptyline	14 (11.0)	2 (6.1)	0.40

DSM-V: Fifth Edition of the Diagnostic and Statistical Manual of Mental Disorders; NRS: numeric rating scale; NSAIDs: non-steroidal anti-inflammatory drugs; SD: standard deviation. * whatever the cause (fibromyalgia, chronic lower back pain or migraine), except tension headaches: more often in dependents (<0.001); ** whatever the cause (inflammatory or cancerous); *** whatever the cause (post chemo, zoosterian, or post trauma).

## Data Availability

All data generated or analyzed during this study are included in this published article.
